# Emergency unit assessment of seven tertiary hospitals in Nepal using the WHO tool: a cross-sectional study

**DOI:** 10.1186/s12245-023-00484-2

**Published:** 2023-02-23

**Authors:** Ramu Kharel, Ghan B. Thapa, Tamara Voor, Samriddha R. Pant, Samir K. Adhikari, Bimal S. Bist, Pryanka Relan, Timmy Lin, Derek Lubetkin, Giovanna Deluca, Olita Shilpakar, Sanu K. Shrestha, Yagya R. Pokharel, Santosh Paudel, Ajay S. Thapa, Yogendra M. Shakya, Achyut R. Karki, Nishant Dhakal, Adam R. Aluisio

**Affiliations:** 1 Department of Emergency Medicine, Warren Alpert Medical School of Brown University, Providence, RI USA; 2 Department of Emergency Medicine and Prehospital Care, Grande International Hospital, Kathmandu, Nepal; 3 UT Southwestern Medical Center, Dallas, TX USA; 4 Department of General Practice and Emergency Medicine, Tribhuvan University Teaching Hospital, Kathmandu, Nepal; 5 Health Emergency Operation Center, Ministry of Health and Population, Kathmandu, Nepal; 6 Department of Health, Graduate School of Education, Tribhuvan University, Kathmandu, Nepal; 7 World Health Organization, Geneva, Switzerland; 8 Department of General Practice and Emergency Medicine, Dhulikhel Hospital - Kathmandu University School of Medicine, Dhulikhel, Nepal; 9 Department of Emergency Medicine, Patan Academy of Health Sciences, Lalitpur, Nepal; 10 National Trauma Center, Kathmandu, Nepal; 11 Department of Emergency Medicine, B & B Hospital, Lalitpur, Nepal; 12 Department of General Practice and Emergency Medicine, Bir Hospital, Kathmandu, Nepal; 13 Department of Pre-Hospital and Emergency Medicine, HAMS Hospital, Kathmandu, Nepal

**Keywords:** Emergency care, Nepal, Global health, Emergency assessment, Hospital care, Acute care

## Abstract

**Background:**

In 2021, the Nepal national emergency care system’s assessment (ECSA) identified 39 activities and 11 facility-specific goals to improve care. To support implementation of the ECSA facility-based goals, this pilot study used the World Health Organization’s (WHO) Hospital Emergency Unit Assessment Tool (HEAT) to evaluate key functions of emergency care at tertiary hospitals in Kathmandu, Nepal.

**Methods:**

This cross-sectional study used the standardized HEAT assessment tool. Data on facility characteristics, human resources, clinical services, and signal functions were gathered via key informant interviews conducted by trained study personnel. Seven tertiary referral centers in the Kathmandu valley were selected for pilot evaluation including governmental, academic, and private hospitals. Descriptive statistics were generated, and comparative analyses were conducted.

**Results:**

All facilities had continuous emergency care services but differed in the extent of availability of each item surveyed. Academic institutions had the highest rating with greater availability of consulting services and capacity to perform specific signal functions including breathing interventions and sepsis care. Private institutions had the highest infrastructure availability and diagnostic testing capacity. Across all facilities, common barriers included lack of training of key emergency procedures, written protocols, point-of-care testing, and ancillary patient services.

**Conclusion:**

This pilot assessment demonstrates that the current emergency care capacity at representative tertiary referral hospitals in Kathmandu, Nepal is variable with some consistent barriers which preclude meeting the ECSA goals. The results can be used to inform emergency care development within Nepal and demonstrate that the WHO HEAT assessment is feasible and may be instructive in systematically advancing emergency care delivery at the national level if implemented more broadly.

**Supplementary Information:**

The online version contains supplementary material available at 10.1186/s12245-023-00484-2.

## Introduction

The World Bank disease control priorities estimate that more than half the deaths and around 40% of the total burden of disease in low- and middle-income countries (LMICs) result from conditions that could be addressed with emergency care [1]. The goal of an emergency care system is to deliver time-sensitive services which extend from pre-hospital care through transport to the emergency department and ensure access to critical medical and operative care when needed. Additionally, emergency care is at the forefront of post-disaster response. Even simple protocols can guide providers to transport patients to appropriate facilities and use formal triage to prioritize care based on clinical need rather than order of arrival, and simple checklists can ensure life-threatening conditions are recognized and the chain of survival improved [2]. Though Nepal’s constitution guarantees state-free emergency care to its citizens, recent national disasters have exposed gaps in emergency care access and infrastructure in the country [1–3]. A less than optimal emergency response was seen in two major disaster events in Nepal: the 2015 Gorkha earthquake, and the peak of the COVID-19 Delta wave in 2021 [4–6]. Given Nepal's high vulnerability to natural disasters, it is imperative to assess the current emergency care capacity and identify areas of improvement [7].

In Nepal, emergency care at different health facilities is provided predominantly by providers without formal training in emergency medicine. Only a handful of emergency medicine-trained specialists (trained via Fellowship or Doctorate of Medicine in Emergency Medicine (DM)) exist in the country. No uniform post-graduate level training in emergency medicine is available at the time of this study [8]. Additionally, there is a dearth of available literature on emergency services available at the hospital level [9].

In 2021, the Nepal Ministry of Health and Population (MoHP) and the World Health Organization (WHO) conducted a stakeholder analysis, the emergency care system assessment (ECSA), which identified 39 key priorities to improve emergency system in the country. Included in these priorities were creating standardized emergency care treatment and transfer protocols in pre-hospital and in-hospital settings [10]. However, a formal evaluation of the ECSA priority areas has not been completed. To fulfill these and other goals identified through the ECSA and to understand emergency care at the facility level, this pilot study systematically evaluates the current status of hospital-based emergency care delivery by assessing emergency units in tertiary hospitals in Kathmandu, Nepal.

## Methods

### Study design and tools

This cross-sectional mixed-method study was conducted using a modified version of WHO’s Hospital unit Emergency Assessment Tool (HEAT). This instrument was developed in 2018 by experts supporting the WHO’s global emergency, trauma, and acute (ETA) program and has been used prior in other countries, including recently in Eswatini [11, 12]. The tool, which has been previously described, includes open-ended, numbered responses, and discrete answers to gather information on facility characteristics, human resources, clinical services, and signal functions [11]. The tool has subsequently been updated since its first design, and given the context of the COVID-19 pandemic during the time of this assessment, additional questions on COVID-19 preparedness derived from the Centers of Disease Control and Prevention (CDC) COVID-19 Comprehensive Preparedness Tool and WHO’s rapid hospital readiness checklist were added to this assessment [13–15]. The tool was reviewed and revised with input from local investigators from Nepal and the Nepal Ministry of Health and Population’s (MoHP) Health Emergency Operation Center (Supplement Material 1). Ethical approval for the study was obtained from LifeSpan Institutional Review Board (IRB), the Nepal Health Research Council, and each individual facility’s Institutional Review Committee. Written informed consent forms were obtained from all respondents participating in the evaluation.

### Facility selection

Kathmandu, the capital city of Nepal, was chosen for the study because of the city's status as the hub of high-level care delivery and the highest patient flow in Nepal. Kathmandu hosts Nepal’s most tertiary care centers, and the city had the highest prevalence of COVID-19 in Nepal during the time of the study selection [6, 16]. Seven Facilities within the region were purposefully chosen in consultation with the MoHP and the Nepal WHO country office, as well as emergency care experts in the country. These facilities were identified as the most highly utilized while serving as a representative sampling of tertiary-level health facilities in the broader context of Nepal. Of the seven hospitals included in the study, two are governmental, three are academic-university affiliated and two are private (Table 1).

**Table 1:** Seven facilities are selected for this study. Facilities are located in or areas surrounding the Kathmandu valley, and include a mix of public, governmental, and academic facilities

Hospital	Location	Category
Bir Hospital	Kathmandu	Public, governmental
Patan Hospital	Lalitpur	Public, Academic-Governmental University
Dhulikhel Hospital	Kavre	Public, Academic-University
Tribhuvan University Hospital (TU)	Kathmandu	Public, Academic-University
Grande Hospital	Kathmandu	Private
HAMS Hospital	Kathmandu	Private
National Trauma Center (NTC)	Kathmandu	Public, governmental

### Respondent selection and data collection

Each hospital had a designated site investigator who coordinated the respondent selection and data collection at their respective facilities. From each hospital of interest, three eligible respondents were identified and interviewed. Respondents were hospital staff members who have been working at the selected hospital for at least three months and are directly involved with the emergency department in a clinical care or administrative leadership role. Questions were reviewed by study staff to determine appropriateness for clinicians (physicians and nurses), administrators (generally, physician and nurse leaders), or general staff, and were asked of each participant based on the determined appropriateness. Each interview took approximately one hour. Three research assistants trained in the study protocol collected data per site visit in hard-copy format. The data was subsequently inputted into a password-protected electronic database.

### Statistical analysis

Descriptive and comparative analysis was conducted using spreadsheet software. The availability rating as well as signal function rating of WHO designated key tasks of the emergency center included an availability scale from 1 to 3, with 1 indicating services that are generally unavailable, 2 indicating some availability, and 3 indicating adequate availability, using definitions in the WHO HEAT tool. For items ranked either 1 or 2, further questions on potential barriers were asked. Rating score was averaged from each section and compared between each type of hospital: governmental vs. teaching vs. private. The rating scale scores from each facility were averaged for final input if the same question was asked to multiple participants, and any discrepancies in discrete or open-ended answers were discussed further with hospital administrators for clarification. An individualized report was made available to each facility, and to the MoHP.

## Results

**Fig. 1:**
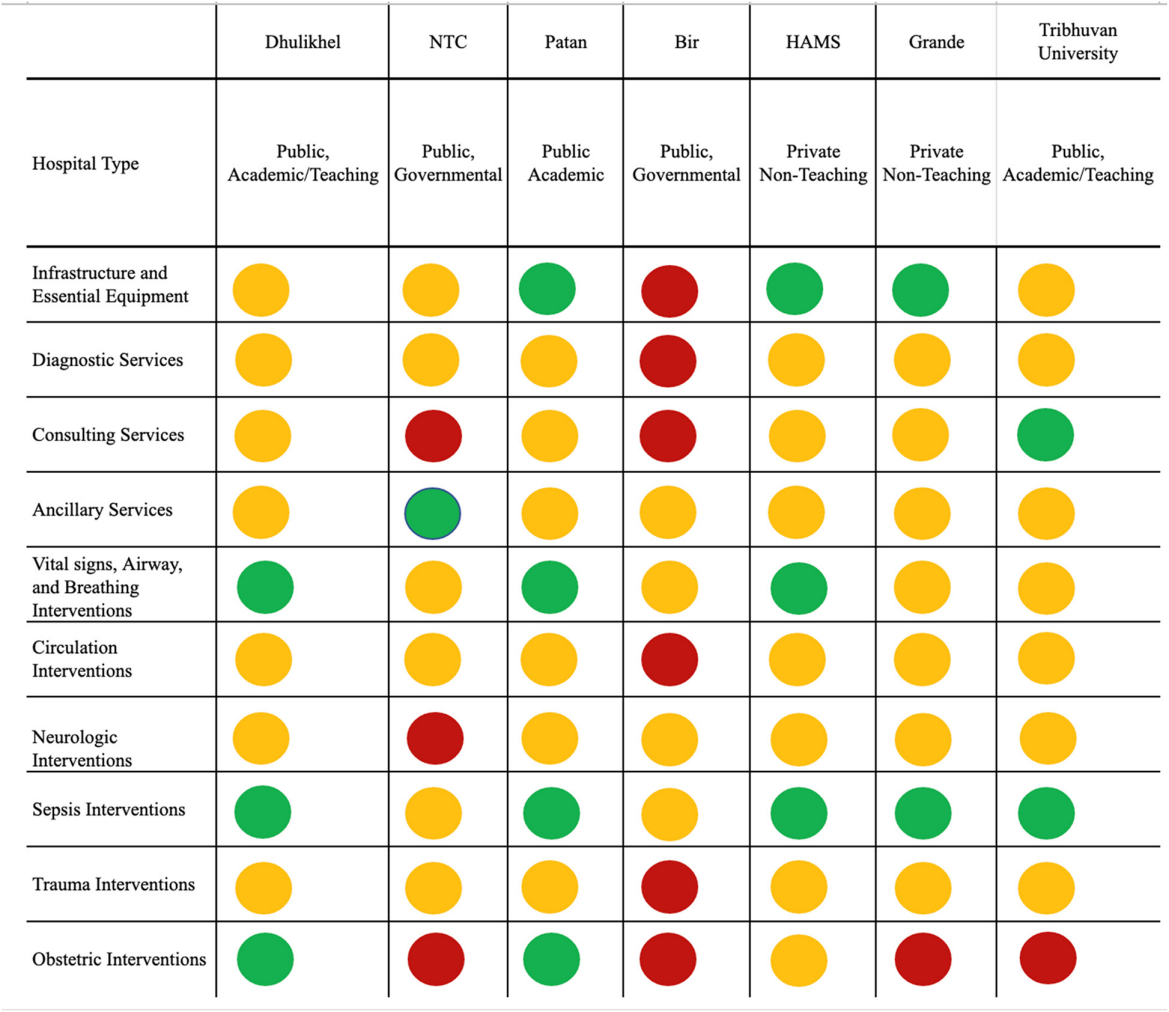
Summary of hospital-specific capacity to support health services and interventions (*n*=7). Percentages are derived from an average score of all items divided by the highest possible score, 3, within each element. Elements with more than 95% of items readily available are depicted with a green circle, some availability (75–95%) is depicted with a yellow circle, and general unavailability (< 75%) is depicted with a red circle

All the facilities surveyed had general availability of 24/7 emergency services and majority of the emergency laboratory testing and diagnostic imaging services. Emergent therapeutics like ventilators, glucose administration, aspirin, antibiotics, vasopressors, and wound care were reported as available at all facilities surveyed. However, there were significant gaps as well as differences in infrastructure, diagnostic, consultant, human resource availability, and signal functions found among facilities. The results presented here highlight findings from each facility with a focus on the gaps and differences in facilities. Figure 1 provides the overall summary of the findings. Detailed description of results from each facility can be supplied by the corresponding author upon request. The COVID-19-related assessment will be presented in future publications.

### Facility characteristics

**Fig. 2:**
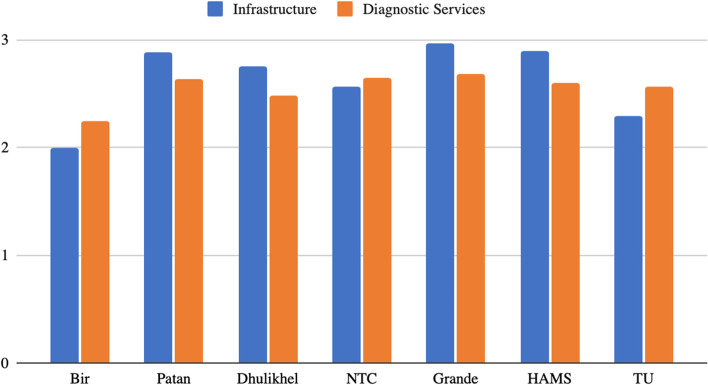
Facility characteristics rating. The graph represents facility characteristics score in rating of 1–3. Private institutions scored highest in infrastructure availability. Bir hospital scored lowest in both infrastructure and diagnostic services availability

Figure 2 presents summary of facility characteristics.

Bir Hospital, a public governmental hospital, is one of the busiest medical centers in Nepal, with 24/7 emergency services available, including MRI availability. On the infrastructure and essential equipment assessment, Bir Hospital received a score of 2.00 out of 3.00, and 2.24 out of 3 on diagnostic services. Notably, the emergency room (ER) did not have a designated triage area or waiting area, nor a designated resuscitation room. Electronic charting was not available. The National Trauma Center (NTC) is a public governmental hospital and the only trauma center in Nepal, with 32 beds for general emergency care and 6 beds for acute resuscitations, and a 24/7 availability of the ER. Emergency overnight MRIs were done at the adjacent Bir Hospital. On the infrastructure and essential equipment and diagnostic services assessment, NTC received a 2.57 and 2.56 out of 3.00, respectively. Limited availability of adequate isolation rooms and hand washing facilities was reported. Point-of-care ultrasound was readily available in the ER.

Patan Hospital is an academic-governmental hospital with nearly 36,000 emergency visits per year and 24/7 availability of emergency services (except for MRI). A separate ER for COVID-19 patients was established during the time of the study. Patan Hospital scored 2.89 on the infrastructure and essential equipment assessment and 2.64 on diagnostic services. Inadequate availability of toilet facility and isolation rooms were reported. Electronic charting was under development during this assessment. Dhulikhel Hospital is a teaching hospital with nearly 20,000 emergency visits per year. The ER has 30 general emergency beds and three acute resuscitation beds with 24/7 emergency service availability (except for MRI). On the infrastructure and essential equipment assessment, Dhulikhel Hospital received a score of 2.76 and on diagnostic services, it scored 2.48. Limited isolation rooms, waiting area space, toilet facilities, and inadequate crash trolley were reported. The ER charting is done in an electronic medical recording system that was recently developed. Tribhuvan University (TU) Teaching Hospital, an academic-semi-governmental hospital (under the Ministry of Education), reported 45,000 emergency unit visits per year, with 24/7 availability of emergency facilities. On infrastructure and essential equipment assessment, Tribhuvan University received a score of 2.29, and on diagnostic services, it scored 2.56. TU reported limited availability of electronic ER charting, adequate isolation rooms for infectious diseases, a designated waiting area, and did not have access to toilet facilities in each patient care area.

Grande Hospital is a private non-teaching hospital with nearly 5,856 emergency unit visits per year and a 24/7 availability of ER services. The ER reported having 15 general emergency care beds and two beds for acute resuscitation. On the infrastructure and essential equipment assessment, Grande received a score of 2.97 and 2.68 for diagnostic services. A lack of adequate isolation beds was reported. HAMS is also a private non-teaching hospital with 4800 emergency unit visits per year, with 17 rooms for general emergency care and 2 for acute resuscitation, and 24/7 availability of emergency services. On the infrastructure and essential equipment assessment, HAMS received a score of 2.90 and 2.60 for diagnostic services. There is no electronic ER charting.

### Human resources

**Fig. 3:**
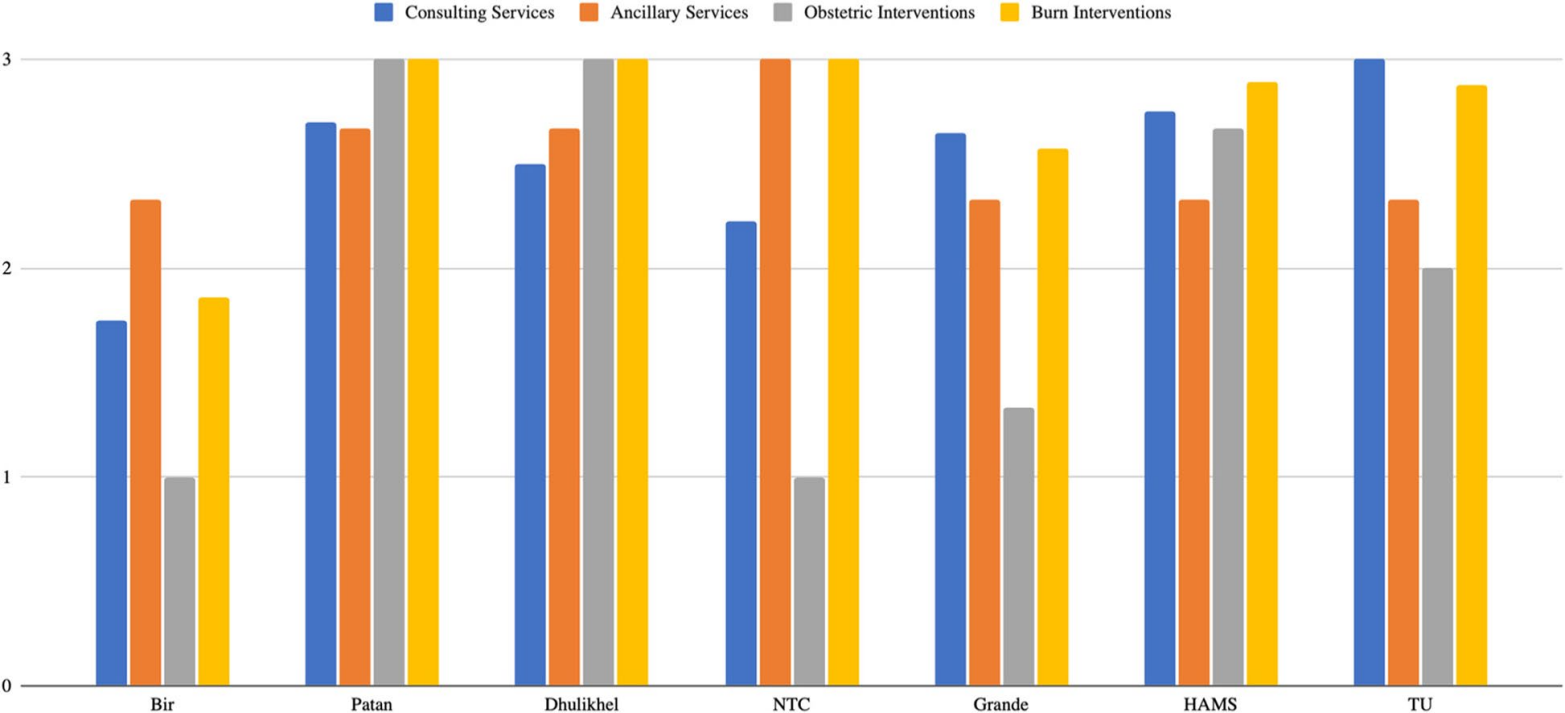
Human resources summary. This demonstrates human resource availability on a scale of 1–3

Figure 3 provides summary of human resources findings.

Out of 3, Bir Hospital scored 1.75 for consulting services and 2.33 for ancillary services. ER was staffed with General Practice (GP) specialists and medical officers primarily. Burn specialists were only available at certain hours, and no Obstetrics/Gynecology (Ob/Gyn), orthopedics, pediatric, or psychiatry consulting services were available in the emergency department. Limited availability of patient transport services and security personnel was also reported. NTC received 2.22 for consulting services and 3.00 for ancillary services. NTC ER was run primarily by staffed medical officers and mid-level providers, with orthopedic specialists at certain times. Notable unavailability included Ob/Gyn providers, pediatricians, psychiatrists, and plastic/reconstructive surgeons. Ancillary services, including social workers, received a high score of 3.00.

Patan Hospital ER received 2.70 for consulting services and 2.67 for ancillary services. The ER staffing included medical officers, GPs, and fellowship-trained emergency medicine (EM) providers. Patan hospital started the first fellowship in EM. Limited availability of burn specialists, plastic/reconstructive surgeons, and patient transport services was reported. Dhulikhel Hospital scored 2.50 for consulting services and 2.67 for ancillary services. The ER staffing included medical officers and at least one GP or fellowship-trained EM provider during a shift. Limited availability of burn and plastic/reconstructive specialists and patient transport service was reported. TU received 3.00 for consulting services and 2.33 for ancillary services. Limited availability of social work services and security personnel assigned to the emergency service area was reported.

Grande Hospital received 2.65 for consulting services and 2.33 for ancillary services. Grande ER is staffed with GPs, EM fellowship/DM-trained physicians, and medical officers. Limited availability of burn and plastic/reconstructive surgeons, ear-nose-throat specialists, neurology, ophthalmology, and social work services were reported. HAMS received a score of 2.75 for consulting service and 2.33 for ancillary service. The ER was staffed with a medical officer and GP specialist or fellowship/DM in EM-trained physician. Limited availability of burn and plastic/reconstructive surgeons, and social work services was reported.

### Clinical services

At Bir Hospital, vital signs are measured on registration, but no formal triage system is reported. Specific clinical management or condition-specific protocols, transfer protocols, and discharge protocols were reported missing. Safety protocols, including infection prevention and post-exposure prophylaxis, were reported available. Quality improvement (QI) is conducted in the ER. Missing aspects of QI include a systematic process for collecting patient data that links conditions and regular meetings for QI. At NTC, 20% of patients were reported to arrive by ambulances with formally trained prehospital providers. A time target for each triage category was reported missing. Most safety protocols were reported available. Absent protocols included transfer, neonatal resuscitation, volume resuscitation of children and adults, adjusting interventions for malnourished patients, and management of labor and delivery in low-risk women. All six condition-specific management protocols asked about were reported missing, including asthma exacerbation, pneumonia, maternal hemorrhage, sepsis, diabetic ketoacidosis, and burn care management. For QI, regular meetings with review of clinical data were reported.

Patan Hospital reported that 20% of patients arrive with formally trained prehospital care providers. Time targets for each triage category and triage protocols for both children <5 years of age and pregnant women were missing. Four of six safety protocols were reported available. Three of five discharge protocols and transfer/referral protocols for burn care were reported missing. In QI, most of the asked questions were reported as available. At Dhulikhel hospital, nearly 35% of emergency patients arrived by ambulance with formally trained prehospital providers. Time targets for each triage category were reported missing. Furthermore, missing protocols for volume and medical resuscitation, burn care, and discharge/transfer were reported. Management of hazardous exposure was also reported missing. Most QI interventions were reported to be conducted except for holding regular meetings using clinical data and tracking to ensure QI actions are implemented after review meetings. At TU, a formal triage system was available, but protocols for triage were reported missing. Time targets for each triage category, triage protocols for children <5 years of age, and triage protocols for pregnant women were reported missing. All condition-specific management protocols in the data were available. Unavailable clinical management protocols included medical resuscitation checklists, neonatal resuscitation, burn care, and adjusting interventions for malnourished patients. Discharge protocols were also reported missing. Most safety protocols, including infection prevention and post-exposure prophylaxis, were available. QI metrics were reported as available.

At Grande, 15–20% of the patients were reported to arrive by ambulance with formally trained prehospital providers. Protocols for time targets in triage, triage for children <5 years of age, trauma care, volume resuscitation, burn care, and all six condition-specific management were reported missing. Most discharge protocols and condition-specific transfer protocols were missing. All QI metrics surveyed were reported as available, except for documentation of supervisor visits with feedback or comments. At HAMS, 15–20% of patients were reported to arrive with formally trained prehospital care providers. Protocols missing were time targets for triage, triage for patients <5 years old or pregnant women, neonatal resuscitation, trauma care, and burn care. Three of six condition-specific protocols were reported to be available. Most discharge and transfer protocols were reported available. Safety protocols are missing, including for managing hazardous exposures and protection of staff and patients from violence.

### Signal functions

**Fig. 4:**
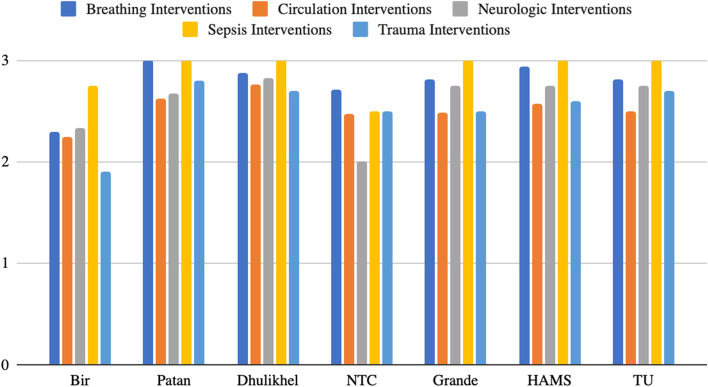
Signal function summary. This presents the availability rating for signal functions from 1 to 3 for each facility

Figure 4 summarizes signal function findings.

Bir Hospital received scores of 2.29 for vital signs/airway/breathing interventions, 2.24 for circulation interventions, 2.33 for neurologic interventions, 2.75 for sepsis interventions, 1.90 for trauma interventions, 1.00 for obstetric interventions, and 1.86 for burn interventions. Notable unavailable interventions included invasive mechanical ventilation in the ED, external defibrillator, training for intraosseous (IO) access, venous cutdown, thrombolytics administration for MI, and training for pelvic binding, point-of-care ultrasound, pericardiocentesis, and external pacing. Unavailable neurological interventions included training for temperature management and mental status exam (MSE), IV magnesium, and equipment for safe physical restraints. trauma, burn, and obstetric cases were transferred quickly to adjacent hospitals, and most of the items on the survey were unavailable. NTC received 2.71 for vital signs/airway/breathing interventions, 2.47 for circulation interventions, 2.00 for neurologic interventions, 2.50 for sepsis interventions, 2.50 for trauma interventions, 1.00 for obstetric interventions, and 3.00 for burn interventions. Bir Hospital ER has limited availability of interventions for breathing and circulation including and is unable to offer the creation of surgical airway, non-invasive ventilation, invasive mechanical ventilation, central venous line placement, pericardiocentesis, thrombolytic administration, IO access, and external defibrillation/cardioversion. Reported unavailable neurological interventions included lumbar puncture (LP), IV magnesium administration for eclampsia, MSE, safe physical restraints, and relevant antidote administration for toxic exposure. Diagnostic paracentesis was reported unavailable for sepsis intervention. Unavailable trauma interventions included three-way dressing for sucking chest wounds due to a lack of training and rabies vaccination or IVIG. Obstetric interventions were mostly reported unavailable. Burn interventions were reported as a score of 3.00, although burn protocols and training were unavailable as reported in an earlier section.

Patan Hospital received a 3.00 for vital signs/airway/breathing interventions, a 2.62 for circulation interventions, a 2.67 for neurologic interventions, a 3.00 for sepsis interventions, a 2.80 for trauma interventions, a 3.00 for obstetric interventions, and a 3.00 for burn interventions. Cardiac pacing and thrombolytic administration for MI were reported as generally unavailable, and IO access, central venous line placement, and pericardiocentesis were reported as somewhat available. For neurologic interventions, extreme temperature management, safe physical restraint, and relevant antidote administration for toxic exposure had limited availability. In trauma care, limited interventions were fasciotomy and rabies vaccinations. Dhulikhel Hospital received a score of 2.88 for vital signs/airway/breathing interventions, 2.76 for circulation interventions, 2.83 for neurologic interventions, 3.00 for sepsis interventions, 2.70 for trauma interventions, 3.00 for obstetric interventions (with a separate birthing center available), and a 3.00 for burn interventions. Limited breathing and circulation interventions included low availability of invasive mechanical ventilation, external cardiac pacing, and thrombolytic administration for MI. For neurological interventions, safe physical restraints had limited availability. There was limited availability of fasciotomy or escharotomy, tetanus, and rabies vaccines or IVIG for trauma interventions. TU received a score of 2.82 for vital signs/airway/breathing interventions, 2.50 for circulation interventions, 2.75 for neurologic interventions, 3.00 for sepsis interventions, 2.70 for trauma interventions, 2.00 for obstetric interventions, and 3.00 for burn interventions. Somewhat unavailable interventions in breathing and circulation included a lack of equipment for placement of oral or nasal-pharyngeal airway device, invasive mechanical ventilation, and a lack of training and surgical personnel for the creation of a surgical airway. Circulation interventions were limited by a lack of equipment for pelvic binding, limited equipment for point-of-care ultrasound, no trained personnel for intraosseous access, pericardiocentesis, or external cardiac pacing, and limited stock and personnel for thrombolytic administration for MI. Of note, a separate cardiac emergency unit is located 100 meters away from the TU ER. Neurological interventions were reported to be limited due to the absence of equipment for protection from secondary injury and safe physical restraint. Trauma interventions were limited by a lack of training and personnel for three-way dressing for sucking chest wound, fasciotomy/escharotomy for compartment syndrome, and limited stock for rabies vaccination and IVIG as appropriate. Obstetric cases were transferred to the labor room 100 meters away. All burn interventions were reported available, however, burn patients were reported to be not kept for 24 h with resuscitation prior to transfer.

Grande Hospital received 2.82 for vital signs/airway/breathing interventions, 2.48 for circulation interventions, 2.75 for neurologic interventions, 3.00 for sepsis interventions, 2.50 for trauma interventions, 1.33 for obstetric interventions, and 2.57 for burn interventions. Breathing and circulation interventions that were limited included creation of surgical airway due to lack of training, IO access and tourniquet placement due to absent equipment, pericardiocentesis, cardiac pacing, and point-of-care ultrasound due to lack of training and equipment. Neurological interventions missing included safe physical restraints and lack of antivenom and certain antidotes. No diagnostic paracentesis was available for sepsis workup in the emergency room. For trauma, three-way dressing for sucking chest wounds, fasciotomy, and reduction of fractures/dislocations were reported to be limited due to lack of training. Obstetric interventions had a low score due to a lack of uterotonic drugs, lack of training in neonatal resuscitation, and limited availability of emergency vaginal delivery due to inadequate training. For burn care, IV fluid resuscitation with hourly adjustments and experience with burn care management was missing. HAMS received 2.94 for vital signs/airway/breathing interventions, 2.57 for circulation interventions, 2.75 for neurologic interventions, 3.00 for sepsis interventions, 2.60 for trauma interventions, 2.67 for obstetric interventions, and 2.89 for burn interventions. For breathing and circulation, limited availability of surgical airways due to lack of training was reported, and limited external cardiac pacing, pericardiocentesis, and thrombolytic administration for MI were reported. For neurological interventions, limited interventions were a lack of extreme temperature management, safe physical restraints, and relevant antidotes for toxic exposures. For trauma, there was a lack of fasciotomy, three-way dressings, and supplies for rabies vaccines or IVIG. For obstetrics, limited availability of vaginal deliveries due to a lack of training and equipment was reported. For burn interventions, fluid resuscitation with hourly adjustments was not done.

## Discussion

This was the first emergency services assessment conducted at tertiary hospitals in Nepal, and the first time the WHO’s HEAT assessment was conducted in Nepal. This pilot study details emergency care capacity in the selected facilities and systematically identifies the strengths of each emergency department and highlights limitations in the emergency care system such as training, resource availability, and human resources. This study provides a unique insight into the emergency unit at major hospitals in Kathmandu and compares the emergency care capacity at private, governmental, and academic hospitals. Furthermore, the study provides specific focus areas needed to improve emergency care at the tertiary centers studied. This study also shows this tool’s feasibility to be used in Nepal’s context. A comprehensive national-level HEAT assessment will be needed to understand emergency care in the country outside of the Kathmandu valley.

Notable features consistent in all EDs were the lack of point-of-care testing for arterial blood gas, carboxyhemoglobin, urine pregnancy, malaria rapid testing, rapid HIV, and urine dipstick. There were some thematic differences between governmental and non-governmental institutions. Bir Hospital and NTC, both governmental facilities, reported better availability of ancillary services and social care services, whereas these services were reported missing at the Dhulikhel, Patan, Grande, and HAMS emergency department. Government facilities generally care for low-income populations and care for a larger volume of patients. Having these ancillary services is likely an important aspect to patient care at the government hospitals. Private hospitals scored highest in infrastructure assessment reflecting on the private sector funding available in building these health care facilities. Overall, written protocols for common conditions, discharge, and transfer were reported missing in most studied facilities. Governmental and private hospitals scored the lowest in the availability of written protocols compared to public academic institutions. Notably, the EDs of TU, Dhulikhel, and Patan reported having more written protocols than other facilities surveyed. Academic hospitals are likely to have more protocols as they conduct more research and teaching compared to non-academic facilities. Consistently, a lack of training or expertise was cited as a reason for the limited availability of certain lifesaving interventions, including the creation of surgical airways, cardiac pacing, pelvic binding, and safe physical restraints. This identifies an acute need for increase in training for these specific emergent procedures.

The findings in this assessment detail some strengths and weaknesses at each of these facilities and highlight an opportunity for inter-institutional collaboration to improve emergency care. For example, Bir Hospital could collaborate with the nearby NTC to build a functional triage system. NTC and Bir are both governmental institutions governed under the same management structure, hence collaboration between these institutions is likely more feasible. Similarly, available written protocols from some facilities could likely be shared and replicated in other facilities through inter-institutional collaboration. This study highlights opportunities for joint skills training in life-saving procedures. Some examples include cardiac pacing, pelvic binding, and burn fluid resuscitation. Dhulikhel Hospital has demonstrated strength in accepting patients from trained prehospital providers (nearly 35% compared to 20% at other facilities), and this institution could lead others in guiding pre-hospital protocols. Dhulikhel ER runs its own emergency medical system (EMS) control center located on the outskirts of Kathmandu to receive the first incomers from outside the valley. Strengthening outgoing and incoming transfer protocols is specifically important for improving care at this institution.

**Fig. 5:**
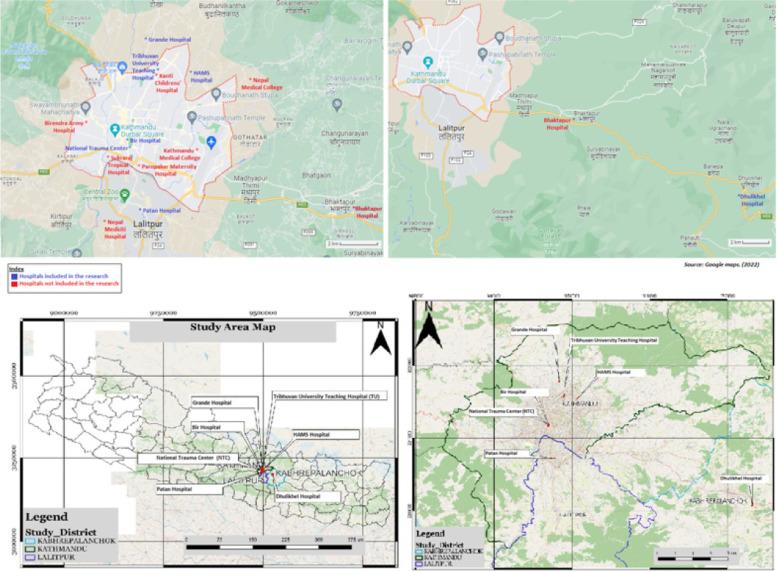
Map of Kathmandu valley and the selected facilities. Other major health facilities near the selected facilities are also highlighted

The results of this study must be taken under the context of the tool used as well. The tool does not allow for us to consider the context of some of these hospitals. For example, some hospitals are located next to specific obstetric and/or trauma hospitals (please look at Fig. 5 for context) hence they do not have services available for these conditions. Additionally, the study tool specifically asks for written protocols, and when facilities report a lack of written protocols, this does not capture non-written established protocols. Nonetheless, the assessed hospitals are considered the highest level facilities in Nepal, where patients from all across the country present to receive care. Many of these facilities also have helicopter-based EMS to quickly receive patients from the remotest parts of the country. Holding these institutions to the highest standard is appropriate as they can set the standard of emergency care in other facilities across the country. This assessment provides a framework for these institutions to address emergency care gaps (Table 2).

**Table 2:** Authors’ recommendations to address identified gaps in emergency care at tertiary facilities in the Kathmandu region are listed in this table

Recommendation 1	Triage system and time metrics	-Ensure all facilities have an adequate triage system in the ER -Ensure time targets in triage -Ensure labeled resuscitation and high acuity areas within the ER
Recommendation 2	Training and education	-Ensure universal emergency staff training and competency with a focus on following procedures: establishment of surgical airway, pelvic binding, cardiac pacing, pericardiocentesis, burn fluid resuscitation, neonatal resuscitation -Consider cross-institutional collaboration in training programs
Recommendation 3	Written protocols for emergency care	-Ensure availability of written protocols for condition-specific care, transfer, and discharge -Ensure cross-institutional collaboration in sharing written protocols
Recommendation 4	Infrastructure	-Ensure minimal standards are met in emergency room infrastructures with a focus on the following: adequate isolation area, waiting area, hand-washing stations, and toilet facilities -Hospital administration must ensure the availability of essential emergency care materials: point-of-care testing, 24/7 radiology, point-of-care ultrasound, etc.

### Limitations

The assessment was conducted in emergency care settings specifically, and the studied metrics may not represent the entirety of healthcare capacity within a given hospital system. This pilot assessment depicts emergency care in urban, highly resourced hospitals in Nepal, and thus the results may not be generalizable to the entire country. Further assessment of emergency care capacity across the country with a focus on rural areas will be important to find areas of improvement in emergency care unique to this setting. This tool has not previously been validated for use in Nepal’s context. This survey was conducted during the second wave of COVID-19 in Nepal, when the country had the highest per capita case rates in the world. As such the information might have reflected the state of emergency care during the acute stressor of the COVID-19 pandemic. This could have contributed to the lack of some services that may be normally available. For example, all the hospitals reported a lack of adequate isolation rooms. Additionally, it is possible that the data could suffer from desirability and responder bias as the survey relies on individual participants' specific experience and knowledge of their facility. The research team was trained in standardized data acquisition to reduce this and inter-respondent triangulation was used to reduce potential bias. Furthermore, any discrepant results in the survey among respondents were discussed with the on-site investigator or hospital-recorded data was looked up to ensure accuracy. To ensure accuracy and updated data was reported, the on-site investigators were provided the results and allowed to update any updates since the study period.

## Conclusion

This pilot assessment provides an unique insight into the state of emergency care at some of the largest hospitals around Kathmandu, Nepal. Strengthening emergency care is recognized as one of the most cost-effective public health interventions and low-cost emergency care interventions are known to save lives, in both disaster and non-disaster settings [17]. This assessment offers a closer look at the emergency care infrastructure, human resources, clinical services, and signal functioning at seven of the largest hospital systems in Kathmandu, Nepal. This assessment highlights the gaps needed to be filled to improve emergency care metrics as defined in the WHO HEAT tool. Furthermore, it provides the national government, multilateral organizations like the WHO, local clinicians, and researchers with specific areas to focus on as Nepal endeavors to improve its emergency care system.

## Supplementary Information

**Additional file 1.** Nepal Emergency Assessment Tool Supplementary Material 1. WHO Emergency Unit Assessment Tool (Modified). Emergency Unit Assessment Tool used for assessing emergency capacity at seven tertiary hospitals in Nepal.

## Data Availability

The datasets used and/or analyzed during the current study are available from the corresponding author on reasonable request.
