# Supplementary material for: Emergency unit assessment of seven tertiary hospitals in Nepal using the WHO tool: a cross-sectional study
Source: Int J Emerg Med. 2023 Feb 23;16:13. doi: 10.1186/s12245-023-00484-2 (PMC9947884; doi:10.1186/s12245-023-00484-2)
Supplement: Supplementary file 1 — Additional file 1. Nepal Emergency Assessment Tool Supplementary Material 1. WHO Emergency Unit Assessment Tool (Modified). Emergency Unit Assessment Tool used for assessing emergency capacity at seven tertiary hospitals in Nepal. [file 12245_2023_484_MOESM1_ESM.pdf]

World Health Organization

EMERGENCY UNIT ASSESSMENT TOOL (Modified)

Table of Contents

Introduction

How to use this tool

Scoring system

Signal Functions

Emergency Unit Assessment

1. Facility Characteristics
   - 1.1 Identifying Information
   - 1.2 Facility Metrics
   - 1.3 Infrastructure and essential equipment
   - 1.4 Diagnostic Services
2. Human Resources
   - 2.1 Emergency Care Clinical Providers
   - 2.2 Consulting services Available to the Emergency Unit
3. Clinical Services
   - 3.1 Access
   - 3.2 Triage
   - 3.3 Guidelines, protocols and checklists
   - 3.4 Ancillary Services
   - 3.5 Quality improvement
4. Signal functions
   - 4.1 Vital Signs
   - 4.2 Airway Interventions
   - 4.3 Breathing Interventions
   - 4.4 Cardiac Interventions
   - 4.5 Neurologic Interventions
   - 4.6 Sepsis Interventions
   - 4.7 Trauma Interventions
   - 4.8 Obstetric Interventions
   - 4.9 Burn Interventions
5. Essential Resources for Emergency Care

Introduction
Emergency care addresses a wide-range of medical, surgical, and obstetric conditions, including injury, complications of pregnancy, exacerbations of non-communicable diseases (e.g. heart attacks, strokes), and acute infections (e.g. sepsis, malaria). Particularly in areas where barriers to care exist, emergency units are often the first point of contact with the healthcare system. Emergency care is a critical component of universal health care, and with sound planning and organization, has the potential to address conditions causing over half of deaths and a third of disability incurred annually in low- and middle-income countries.

A strategic assessment of emergency care capacity at healthcare facilities is among the first steps in the planning process. Findings can be used to identify gaps and target interventions at both individual facilities and across the healthcare system more broadly; in addition, periodic assessments may also be useful for monitoring capacity over time.

This assessment tool designed to evaluate the structure and key functions of an emergency unit (or any dedicated intake area for acutely ill and injured patients). It is derived from the WHO *Emergency Care System Framework*, WHO *Guidelines for Essential Trauma Care*, WHO *Tool for Situational Analysis to Assess Emergency and Essential Surgical Care* and the African Federation for Emergency Medicine *Emergency Care Assessment Tool*, and was informed by a broad review of other relevant instruments.

How to use this tool
This tool is designed to assess emergency care capacity and organization at the facility level. It can be used at an individual facility or across a group of facilities region- or country-wide. Hospital administrators and health system planners can use the findings to identify gaps in order to guide planning. If the tool is to be used at the regional or country level, either all facilities within a certain category should be assessed (all regional or district hospitals, for example), or a robust sampling strategy should be utilized to ensure that the facilities assessed best represent the regional or national reality. Sampling strategies for facility-based assessments include:

- Exhaustive sampling –all facilities in the target area (e.g., region, country) or of a specific type are assessed (eg. all district hospitals).
- Random sampling (including stratified and cluster random sampling) – facilities to be assessed are randomly selected from among a target group
- Purposeful sampling – facilities are chosen by well-informed stakeholders specifically to reflect the diversity of facilities, geography, administrative areas, patient volume, levels of care, etc.;

Exhaustive sampling of all relevant facilities is the most robust approach, but may not always be feasible. Considerations for choosing a sampling strategy include: objectives of the assessment, needs of stakeholders, total number and types of facilities, heterogeneity of the country or region (differences between urban and rural areas, for example), and need for statistical certainty. For more details on sampling strategies and sample size calculations for facility-based capacity assessments, see the WHO *Service Availability and Readiness Assessment* (SARA): http://www.who.int/healthinfo/systems/SARA_Implementation_Guide_Chapter2.pdf.

Prior to performing the assessment, permission should be sought and granted by the appropriate national agencies and facility administrators. The facility administration can also help identify key informants to participate in the assessment. Ideally, more than one key informant is approached to complete the tool to allow triangulation of responses, which may improve the accuracy of the results. If more than one key informant completes all or the respective part of the tool, there are multiple ways to compile the results: i) the median response for each question becomes the final response; or ii) the response of the key informant with the most understanding of a specific resource, service or function becomes the final response; or iii) a consensus-process is initiated (eg, a meeting convened) and a single answer for each question agreed upon by respondents.

Potential key informants that might provide important information for this assessment include:
- Facility administrators (e.g., medical director, human resources director, operations officer, nurse matron);
- Providers (e.g., nurses, clinical officers, specialists) who work in the emergency unit;
- Laboratory and radiology unit technicians;

- Technicians and biomedical engineers who interact with equipment in the aforementioned units/departments;
- Procurement and medical stores staff;
- Facility statistics and health information staff.

Note that not all facilities will have staff in all of these positions, or even have each of the units/departments above. In addition, the informant with the highest authority at the facility may not be the informant with the most accurate understanding of the availability of a given resource. For the section on signal functions, the key informant should someone with direct involvement in clinical care delivery.

It should also be noted that this tool does not define a minimum standard for every emergency unit at every level of the health system. It is intended as a general tool to identify gaps that can be addressed by implementation of standards promoted elsewhere. Ultimately, countries will need to determine which services they aim to provide at a given level of the health system.

Scoring system

There are four question types in this assessment:

1. Open-ended (e.g., name of facility);
2. Number response (e.g., number of emergency unit visits per year);
3. Discrete answers (e.g., yes or no);
4. Availability rating.

The availability rating questions are used to assess resource and service capacities, specifically the ability to perform key functions in the time frame needed for emergency care. These questions are meant to reflect the demand-side factors (e.g., number of patients in need) for the service, as well as the supply-side factors (e.g., sufficient resources, satisfactory training). For each of these questions, the resource or service should be noted as:

1 - Generally unavailable;
2 - Somewhat available (available to **ONLY SOME** of those who need it);
3 - Adequate (**PRESENT and AVAILABLE** to almost everyone in need, and used when needed).

If the availability rating is less than 3 (less than adequate), it is important to know the factors that contribute to its deficiency. Common factors that contribute to inadequate resources (such as supply chain problems, or lack of training) can then be identified and addressed. Therefore, for ratings less than 3, the person administering the survey should systematically prompt the key informant to identify reasons for the less than adequate rating; more than one factor can be marked per resource, service or function.

- *Infrastructure* - physical space, electricity, water
- *Absent equipment* – the resource is not present at the facility
- *Broken equipment* –the resource is present, but not in working order
- *Stock out* – the resource or function cannot be procured, or required supplies out of stock often due to stock management practices or procurement failures (e.g., reagents, tubes, IV catheters)
- *Training* – staff knowledge/skill gaps limit capacity to use the resource or perform a function
- *Personnel* - resource, service or function available, but lack of adequate numbers of staff limit capacity
- *User fees* - resource or function available, but out-of-pocket payment requirement prevents care delivery
- *Opening hours* - hours the facility can be accessed by acute patients
- *Other* – enter explanation in comments field

Signal Function Performance

Emergency care is a cross-cutting, service delivery innovation providing timely intervention for acute conditions such as sepsis or trauma. The capacity to perform key time-dependent interventions for these sentinel conditions can be used as a marker of overall emergency unit performance. Any limited availability of these key interventions signals a critical gap in emergency care delivery capacity. Use of these “signal functions” allows for a rapid, simple assessment and the identification of failures of emergency care delivery whose cause can

then be identified and addressed. For example, identifying limited availability of intravenous volume resuscitation should prompt evaluation for causes such as lack of functioning equipment, supply stock-outs, gaps in staff skills/knowledge, or poor infrastructure.

Emergency Unit Assessment

1. Facility Characteristics

1.1 Identifying Information

1.1.1	**Date**	
1.1.2	**Country**	
1.1.3	**Name of facility**	
1.1.4	**Address of facility** (include city, state or province)	
1.1.5	**GPS Reading** (if available)	Degrees Minutes Seconds<br>Latitude:<br>Longitude:
1.1.6	**Name** person filing out form	
1.1.7	**Facility Contact(s)**	**1. Name:** **Phone:** **Email:**<br>**2. Name:** **Phone:** **Email:**
1.1.8	**Level of facility***	□ Health centre or clinic(1) □ 1st level hospital(2) □ 2nd level hospital(3) □ Tertiary hospital(4)
1.1.9	**Type of facility**	□ Private hospital(1) □ NGO hospital(2) □ Government hospital(3)
1.1.10	Distance to nearest higher level facility:	
1.1.11	Is there an area (room, unit, department) specifically designated for emergency care? **Yes(1)** **No (2)**	
1.1.12	Population served by facility (e.g., 123,000):	

*Footnote: see reference definitions

1.2 Facility Metrics

Descriptor		Number
1.2.1	Emergency unit visits per year - total	
1.2.2	Emergency unit visits per year – children aged ≤ 5 years	
1.2.3	Outpatient visits per year (excluding emergency unit visits)	
1.2.4	Inpatient admissions per year	
1.2.5	Beds/gurneys dedicated for general emergency care (not including inpatient beds)	
1.2.6	Beds/gurneys dedicated to resuscitation of the sickest patients	
1.2.7	Inpatient hospital beds	
1.2.8	Functioning operating theatres (24/7)	
1.2.9	Functioning high acuity unit (e.g. ICU) beds with capacity for continuous monitoring and mechanical ventilation	
1.2.10	Emergency operations per year	

**Available hours**		
1.2.11	During which hours is the emergency unit covered by providers who are physically present in the unit?	
1.2.12	During which hours is the emergency unit covered by providers who are on call, inside the facility?	
1.2.13	During which hours is the emergency unit covered by providers who are on call outside the facility?	
	Opening hours of:	
1.2.14	Emergency Unit	
1.2.15	Laboratory	
1.2.16	Pharmacy	
1.2.17	Radiology	
1.2.18	Operating Theatre	
1.2.19	**Comments:**	

1.3 Infrastructure and essential equipment

Rating: 1 - Generally unavailable, 2 - Some availability, 3 – Adequate

Infrastructure Element		Rating (1-3)	Comments (if rating <3)
1.3.1	Clean, running water		
1.3.2	Electricity source (e.g., wired, generator)		
1.3.3	Designated telephone or radio for communicating with other facilities and/or prehospital providers		
1.3.4	Paper-based emergency unit chart		
1.3.5	Electronic emergency unit chart		
1.3.6	Isolation room for infectious diseases (e.g., TB, haemorrhagic fever)		
1.3.7	Easy physical access to emergency unit for those requiring a wheelchair or stretcher		
1.3.8	Designated waiting area		
1.3.9	Designated triage area		
1.3.10	Designated resuscitation area		
1.3.11	Personal protective equipment (e.g., hair covers, eye protection, N95 face masks, impermeable gowns, shoe covers, gloves) in a range of sizes		
1.3.12	Electronic cardiac monitoring in emergency unit		
1.3.13	Electrocardiograph available in emergency unit		
1.3.14	Crash trolley or code cart with high-acuity equipment and supplies of various sizes in emergency unit		
1.3.15	Rapid access to a transport ambulance and provider to administer care during transport for patients who need to be transferred to another facility		
1.3.16	Is there access to storage space within (or with immediate proximity to) the emergency unit, including secure storage for controlled substances?		
1.3.17	Access to dedicated staff work area (e.g. for paperwork, consultation calls)		
1.3.18	Access to toilet facilities for patients and staff		
1.3.19	Access to handwashing facilities in each patient care area		
1.3.20	System for stocking, managing, and dispensing medications in emergency unit		
1.3.21	Oxygen in emergency unit		

Which of the following methods supply oxygen in this unit?		Yes	No
1.3.22	Oxygen is supplied through a central piped system	1	2
1.3.23	Oxygen is supplied in tanks that are stored on this unit	1	2
1.3.24	Oxygen is supplied by oxygen concentrator stored on this unit	1	2
1.3.25	Emergency unit calls for tank of oxygen from central location if needed	1	2
1.3.26	Emergency unit calls for oxygen concentrator from central location if needed	1	2
1.3.27	**Comments:**		

1.4 Diagnostic Services

Rating: 1 - Generally unavailable, 2 - Some availability, 3 – Adequate (For rating <3, mark all relevant barriers)
[For data entry: code any marked barriers as 1, unmarked barriers as 2]

**Descriptor**		***Rating (1-3)***	*Infrastructure*	*Absent Equipment*	*Broken Equipment*	*Stock out (Supplies)*	*Training*	*Personnel*	*User fees*	*Opening hours*	*Other (specify in comments)*
**Laboratory-based Testing**											
1.4.1	Haemoglobin										
1.4.2	Full blood count										
1.4.3	Coagulation profile (PT/PTT)										
1.4.4	Electrolytes										
1.4.5	BUN and creatinine										
1.4.6	Lipase										
1.4.7	Cardiac marker (e.g., troponin)										
1.4.8	Arterial blood gas										
1.4.9	Cross matching for blood and blood products										
1.4.10	Blood cultures										
1.4.11	Capacity to obtain sterile blood samples for lab testing										
1.4.12	System for reporting urgent lab results in a timely fashion										
1.4.13	System for reporting critical lab results in a timely fashion										
1.4.100	*Blood carboxyhaemoglobin level assessment*										
**Point of Care Testing – available in the emergency unit**											
1.4.14	Urine dipstick										
1.4.15	Urine pregnancy										
1.4.16	Glucose										
1.4.17	Malaria Rapid Diagnostic Test (RDT)										
1.4.18	Rapid HIV testing										
**Diagnostic imaging**											
1.4.19	Stationary X-ray										
1.4.20	Portable X-ray for use in emergency unit										
1.4.21	Ultrasound in the hospital										
1.4.22	Ultrasound for use in emergency unit										

1.4.23	CT scan									
1.4.24	System for reporting radiology results in a timely fashion									
1.4.25	**Comments:**									

2. Human Resources

2.1 Emergency Care Clinical Providers

2.1.1	Do you have a core of fixed (non-rotating) providers permanently assigned to the emergency unit?	Yes (1)	No (2)

Descriptor		Total Number	Number of licensed or certified
**Number of non-rotating providers assigned to emergency unit**			
2.1.2	Nurses/nurse midwives		
2.1.3	Mid-level provider or advance practice nurses (e.g., clinical officers or nurse practitioners)		
2.1.4	Medical officers (doctors without specialist training)		
2.1.100	*Medical officers with training in acute burn stabilization*		
2.1.5	Emergency medicine specialists		
2.1.101	*Emergency medicine specialist with training in acute burn stabilization*		
2.1.6	Other specialist doctor		
**Number of rotating providers assigned to emergency unit**			
2.1.7	Nurses/nurse midwives		
2.1.8	Mid-level provider or advance practice nurses (e.g., clinical officers or nurse practitioners)		
2.1.9	Medical officers (e.g., doctors without specialist training)		
2.1.103	*Medical officers with training in acute burn stabilization*		
2.1.10	Emergency medicine specialists		
2.1.104	*Emergency medicine specialist with training in acute burn stabilization*		
2.1.11	Other specialist doctor		
2.1.12	**Comments:**		

2.2 Consulting Services Available to the Emergency Unit

Consulting Service		Rating (1-3)	Comments
2.2.1	General Surgery		
2.2.2	OB/GYN		

2.2.3	Orthopaedics		
2.2.4	Anaesthesia		
2.2.5	Paediatrics		
2.2.6	Psychiatry		
2.2.100	*Burn Specialist*		
2.2.101	*Plastic and reconstructive surgeon*		
2.2.7	Other (Please list):		

Rating: 1 - Generally unavailable, 2 - Some availability, 3 – Always available

2.3 Ancillary Services available to the emergency unit

Rating: 1 - Generally unavailable, 2 - Some availability, 3 – Always available

**Ancillary Service**		**Rating (1-3)**	**Comments**
2.3.1	Social work services		
2.3.2	Patient transport services (personnel with wheelchairs and/or gurneys)		
2.3.3	Security personnel assigned to emergency service area		

3. Clinical Services

3.1 Access

3.1.1	What proportion of patients with emergency conditions are brought to the facility by ambulance with formally trained prehospital care providers?	_____ %	Don't know
3.1.100	*What proportion of patients with burns are brought by ambulance with formally trained prehospital care providers?*	*_____ %*	*Don't know*
3.1.2	Are there regulations and/or protocols mandating that acutely ill or injured patients are clinically triaged prior to being required to register?	Yes(1)	No(2)
3.1.3	Does the facility require payment prior to provision of initial emergency care?	Yes(1)	No(2)
3.1.4	Is there an electronic system for registration?	Yes(1)	No(2)
3.1.101	*Are there regulations and/or protocols mandating clinical triage prior to registration for burn-injured patients?*	*Yes(1)*	*No(2)*
3.1.102	*Does facility require payment prior to providing initial burn care?*	*Yes(1)*	*No(2)*
3.1.5	**Comments:**		

3.2 Triage

		Yes	No
3.2.1	Are vital signs measured in triage area?	1	2
3.2.2	Does this facility use a formal triage system (includes a structured triage tool, such as the WHO-ICRC integrated triage tool, used by trained personnel)? If no triage protocols, tick box and skip to 3.3 []	1	2
3.2.3	Are there time targets for each triage category (e.g., YELLOW – seen by provider within 2 hours)?	1	2
3.2.4	If there are time targets, is compliance tracked regularly?	1	2
3.2.5	Are there specific triage protocols for children <5 years of age?	1	2
3.2.6	Are there specific triage protocols for pregnant women?	1	2
3.2.7	**Comments:**		

3.3 Guidelines, protocols and checklists

Are the following written protocols available at this facility? [] No written protocols<br>(if no written protocols in the unit, tick box above and go directly to section 3.4)		Yes	No
3.3.1	Protocol for systematic triage that ensures patients are seen in order of acuity	1	2
3.3.2	Syndromic surveillance guidelines with links to public health officials for case definition and reporting	1	2
3.3.3	Clear protocol for communication with hospital administration during times of overcrowding	1	2
3.3.4	Emergency unit specific emergency response protocol, including protocol for mass casualty incidents	1	2
**Are the following clinical management protocols available at this facility?**			
3.3.5	Protocol for initial approach to ABCDs (airway, breathing, circulation, basic neurologic function)	1	2
3.3.6	Trauma care checklist	1	2
3.3.7	Medical resuscitation checklist	1	2
3.3.8	Protocol for neonatal resuscitation	1	2
3.3.9	Protocol for volume resuscitation of children and adults	1	2
3.3.103	*Burn care checklist*	*1*	*2*
3.3.10	Protocol for adjusting interventions for malnourished patients	1	2
3.3.11	Protocol for post-exposure prevention of STI/HIV, emergency contraception, counselling	1	2
3.3.12	Protocol for management of labour and delivery in low risk women	1	2
**Condition-specific management protocols for:**			
3.3.13	Asthma exacerbation	1	2
3.3.14	Pneumonia	1	2
3.3.15	Maternal haemorrhage	1	2
3.3.16	Sepsis	1	2
3.3.17	Diabetic ketoacidosis	1	2
3.3.104	*Burn care management*	*1*	*2*
3.3.18	Other: ________________________	1	2
**Are the following admission or discharge protocols available at this facility?**			
3.3.19	Acuity-based internal transfer protocols to OT or ICU	1	2
3.3.20	Protocol for timely disposition from the emergency unit	1	2

3.3.21	Protocol for conveying information about discharge or disposition to the patient	1	2
3.3.22	Hand-over protocols when transferring patients from one care provider to another	1	2
**Are the following outside transfer protocols available at this facility?**			
3.3.23	Condition-specific transfer or referral protocols<br>(e.g., criteria for transfer of burn patient to burn centre)	1	2
3.3.24	Communication with receiving facility prior to transfer of patients with emergency conditions	1	2
3.3.105	*Condition-specific transfer/referral protocols for transfer of burn patients to burn centre*	1	2
3.3.106	*Communication with receiving facility prior to transfer of patients to burn centre*	1	2
3.3.107	*Process to provide documentation of initial assessment, treatments provided, and condition on transfer to burn centre*	1	2

**Are the following safety protocols available at this facility?**		**Yes**	**No**
3.3.25	Infection prevention and control protocols	1	2
3.3.26	Protocol for post exposure prophylaxis for health care workers	1	2
3.3.27	Security protocols to protect staff, patients, and infrastructure from violence.	1	2
3.3.28	Protocol for managing hazardous exposures (including designated decontamination area)	1	2
3.3.29	Containment and disposal of sharps and biomedical waste	1	2
3.3.30	Plan to ensure emergency unit staff and patient safety if an incident occurs *within* the emergency unit (including space, transport, communications)	1	2
3.3.31	**Comments:**		

3.4 Quality improvement in the emergency unit

Are the following conducted in the emergency unit?		Yes	No
3.4.1	Systematic process for collecting patient data that links condition, management and outcomes (e.g., trauma registry)	1	2
3.4.2	Regular meetings convened to use clinical data for quality improvement (e.g., morbidity and mortality conferences, preventable death panels)	1	2
3.4.3	Tracking (e.g., clinical audit) to ensure that quality improvement actions (e.g., corrective action) are implemented after review meetings	1	2
3.4.4	Clinical document template (e.g., standardized clinical chart)	1	2
3.4.5	Has there been a visit to this emergency facility by a supervisor from outside the facility within the last 6 months?	1	2
3.4.6	Is there any documentation from the most recent external supervisory visit?	1	2
3.4.7	Does the document provide any feedback or comments on some aspect of emergency services?	1	2
3.4.8	**Comments:**		

4. Signal Function Performance

(The key informants for this section should be personnel with direct involvement in clinical care delivery)
Rating: 1 - Generally unavailable, 2 - Some availability, 3 – Adequate (For rating <3, mark all relevant barriers)
[For data entry: code any marked barriers as 1, unmarked barriers as 2]

VITAL SIGNS, AIRWAY & BREATHING INTERVENTIONS		***Rating*** *(1-3)*	*Infrastructure*	*Absent Equipment*	*Broken Equipment*	*Stock out (Supplies)*	*Training*	*Personnel*	*User fees*	*Opening hours*	*Other (specify in comments)*
**Vital Signs**											
4.1.1	Are vital signs measured in the triage area?										
4.1.2	Are vital signs measured in the Emergency Unit?										
**Airway Interventions**											
4.2.1	Use of manual manoeuvres (e.g., jaw thrust, chin lift)										
4.2.2	Use of suction										
4.2.3	Placement of oro- or naso-pharyngeal airway device										
4.2.4	Placement of supraglottic device (e.g., LMA)										
4.2.5	Endotracheal intubation										
4.2.6	Creation of surgical airway										
**Breathing Interventions**											
4.3.1	Measurement of oxygen saturation at triage										
4.3.2	Measurement of oxygen saturation in emergency unit treatment area										
4.3.3	Administration of bronchodilator for reactive airway disease										
4.3.4	Administration of oxygen										
4.3.5	Bag-valve-mask ventilation										
4.3.6	Non-invasive mechanical ventilation (BiPAP, CPAP)										
4.3.7	Invasive mechanical ventilation										
4.3.8	Needle decompression of tension pneumothorax										
4.3.9	Placement of chest tube										
4.3.10	**Comments:**										

Rating: 1 - Generally unavailable, 2 - Some availability, 3 – Adequate (For rating <3, mark all relevant barriers)
[For data entry: code any marked barriers as 1, unmarked barriers as 2]

CIRCULATION INTERVENTIONS		***Rating*** (1-3)	*Infrastructure*	*Absent Equipment*	*Broken Equipment*	*Stock out (Supplies)*	*Training*	*Personnel*	*User fees*	*Opening hours*	*Other (specify in comments)*
**Volume Resuscitation**											
4.4.1	Administration of oral rehydration										
4.4.2	Peripheral IV placement										
4.4.3	Intraosseous access										
4.4.4	Venous cutdown										
4.4.5	Central venous line placement										
4.4.6	IV fluid administration										
4.4.7	Adjustment of fluid resuscitation for malnutrition or severe anaemia										
4.4.8	Urinary catheter placement										
**Control of Bleeding**											
4.5.1	External control of haemorrhage										
4.5.2	Wound packing and/or suture placement to control bleeding										
4.5.3	Tourniquet placement										
4.5.4	Pelvic binding placement										
4.5.5	Safe transfusion (e.g., including screened blood, maintenance of sterility, monitoring)										
4.5.6	Point of care ultrasound (performance and interpretation)										
**Cardiac Interventions**											
4.6.1	Pericardiocentesis										
4.6.2	External defibrillation and/or cardioversion										
4.6.3	External cardiac pacing										
4.6.4	Adrenaline administration										
4.6.5	ECG with interpretation										
4.6.6	Aspirin administration for ischemia										
4.6.7	Thrombolytic administration for MI										
4.6.8	**Comments:**										

Rating: 1 - Generally unavailable, 2 - Some availability, 3 – Adequate (For rating <3, mark all relevant barriers)
[For data entry: code any marked barriers as 1, unmarked barriers as 2]

**NEUROLOGIC INTERVENTIONS**		***Rating*** *(1-3)*	*Infrastructure*	*Absent Equipment*	*Broken Equipment*	*Stock out (Supplies)*	*Training*	*Personnel*	*User fees*	*Opening hours*	*Other (specify in comments)*
**Unconscious patient**											
4.7.1	Point of care glucose testing										
4.7.2	Glucose administration for hypoglycaemia										
4.7.3	Lumbar puncture										
**Seizure**											
4.7.5	Protection from secondary injury										
4.7.6	Benzodiazepine administration										
4.7.7	IV magnesium administration (for eclampsia)										
**Other**											
4.7.8	Mental status examination										
4.7.9	Extreme temperature management (hyper- or hypothermia)										
4.7.10	Safe physical restraint										
4.7.11	Medication administration for agitation										
4.7.12	Procedural sedation										
4.7.13	Relevant antidote administration for toxic exposure (eg, atropine, naloxone, anti-venin).										
	**Comments:**										

**SEPSIS INTERVENTIONS**		***Rating*** *(1-3)*	*Infrastructure*	*Absent Equipment*	*Broken Equipment*	*Stock out (Supplies)*	*Training*	*Personnel*	*User fees*	*Opening hours*	*Other (specify in comments)*
4.8.1	IV antibiotic administration										
4.8.2	IV vasopressor administration										
4.8.3	Diagnostic paracentesis										
4.8.4	Bedside minor surgical techniques for infectious source control (e.g., abscess)										
	**Comments:**										

Rating: 1 – Generally unavailable, 2 – Some availability, 3 – Adequate (For rating <3, mark all relevant barriers)
[For data entry: code any marked barriers as 1, unmarked barriers as 2]

**TRAUMA INTERVENTIONS**		***Rating*** *(1-3)*	*Infrastructure*	*Absent Equipment*	*Broken Equipment*	*Stock out (Supplies)*	*Training*	*Personnel*	*User fees*	*Opening hours*	*Other (specify in comments)*
4.9.1	Cervical spine immobilization										
4.9.2	Three-way dressing for sucking chest wound										
4.9.3	Fasciotomy or escharotomy for compartment syndrome										
4.9.4	Opiate analgesia administration										
4.9.5	Fracture immobilization										
4.9.6	Closed reduction of fracture or dislocation										
4.9.7	Antibiotic administration for open fracture										
4.9.8	Initial wound care										
4.9.9	Tetanus vaccination or IVIg as appropriate										
4.9.10	Rabies vaccination or IVIg as appropriate										
	**Comments:**										

**OBSTETRIC INTERVENTIONS**		***Rating*** *(1-3)*	*Infrastructure*	*Absent Equipment*	*Broken Equipment*	*Stockout (supplies)*	*Training*	*Personnel*	*User fees*	*Opening hours*	*Other (specify in comments)*
4.10.1	Emergency vaginal delivery										
4.10.2	Uterotonic drug (e.g., oxytocin) administration										
4.10.3	Neonatal resuscitation										
	**Comments:**										

**Burn Care Intervention**		***Rating*** *(1-3)*	*Infrastructure*	*Absent Equipment*	*Broken Equipment*	*Stockout (supplies)*	*Training*	*Personnel*	*User fees*	*Opening hours*	*Other (specify in comments)*
4.11.100	First aid and initial wound care for burn injured patients										

4.11.101	Administration of oral rehydration for burn injured patients									
4.11.102	IV fluid administration per Parkland formula for burn injured patients									
4.11.103	IV fluid resuscitation with hourly adjustments to fluid for burn injured patients									
4.11.104	Urinary catheter placement for burn injured patients									
4.11.105	Nasogastric tube placement in burn injured patients									
4.11.106	Wound assessment including % Total Body Surface Area (TBSA) estimate for burn injuries									
4.11.107	Do you keep burn patient for 24 hours with resuscitation prior to transfer? (1 =Y, 2=N)									
	**Comments:**									

See also: WHO Essential Resources for Emergency Care: Equipment and Supplies

**5**	**COVID Supplemental Questions**				
5.1.100	How many COVID patients have been taken care of at your ER till date?				
	**Leadership and Coordination**	YES	NO	I Don't Know	In Process
5.1.101	Is there developed contingency plans for staffing, logistics, budget, procurement, security and treatment?				
	**Operational support and Logistics**	YES	NO	I Don't Know	In Process
5.1.102	Is there a mechanism for the prompt maintenance and repair of all equipment required for essential services?				
5.1.103	Is there a procedure for managing ambulances for transportation between hospitals and for the inventory of available vehicles?				
5.1.104	Is there a procedure to protect ambulance crew and disinfect ambulance vehicles and equipment after each use?				
5.1.105	Is there availability of appropriate back-up arrangements for essential lifelines, including water, power and oxygen?				
	**Information**	YES	NO	I Don't Know	In Process
5.1.106	Is there a standardized form for reporting on emergency activities, hospitalizations (including critical care), incidence of suspected and confirmed cases, clinical situation and deaths?				
	**Communication**	YES	NO	I Don't Know	In Process
5.1.107	Is the hospital staff briefed on their roles and responsibilities in managing COVID-19 under the Incident Management System?				
5.1.108	Is there regular communication with staff and stakeholders about clinical triage, patient prioritization and management (e.g. adapted admission and discharge criteria), infection prevention and control measures, hospital epidemiology, reporting requirements and security measures?				
	**Human Resources**	YES	NO	I Don't Know	In Process

5.1.109	Are human resources adapted to ensure adequate staff capacity and continuity of operations in response to an increased demand for human resources, while maintaining services identified as essential?				
5.1.110	Are there policies in place to manage volunteer workers (vetting, accepting, rejecting, addressing liability issues, etc.)?				
5.1.111	Is a clear policy established to monitor and manage staff suspected or confirmed of having COVID-19 or who have been exposed to a confirmed, probable or suspected COVID-19 patient?				
5.1.112	Is training and education considered on clinical management, use of personal protective equipment and the handling and disposal of contaminated waste?				
	**Essential Services and Surge Capacity**	YES	NO	I Don't Know	In Process
5.1.113	Are areas identified that can be used to increase patient care capacity (surge capacity) (e.g. use of hospital corridors, lobby and other non-essential spaces and also parking areas and open spaces as a last resort), bearing in mind the necessary physical space, staff, supplies and processes?				
	**Rapid Identification**	YES	NO	I Don't Know	In Process
5.1.114	Is there a triage space established with optimal conditions for the prevention and control of infections to triage patients with acute respiratory symptoms (isolation area for suspected patients, availability of PPEs, disposal of contaminated supplies and linens, etc.)?				
5.1.115	Are signs and information displays provided at the entrance and in waiting rooms regarding Q&As about COVID-19, hand hygiene and respiratory hygiene?				
5.1.116	Is there an established triage protocol aimed at ensuring that cases of acute respiratory infection are rapidly recognized and reported?				
5.1.117	Are health workers trained to identify suspected cases rapidly and accurately in accordance with standardized case definitions, to enable timely reporting to the corresponding level in any area of the hospital?				
5.1.118	Are there alternative triage protocols, for example telephone triage in which the patient needs to call first before going to hospital?				
	**Isolation and Case Management**	YES	NO	I Don't Know	In Process
5.1.119	Are guidelines/protocols provided for the management of suspected or confirmed cases and ensure they are correctly followed?				
5.1.220	Are there trained staff and equipment available for the initial and continued medical care of suspected or confirmed patients (primary screening, resuscitation, initial stabilization, mechanical ventilation and hospitalization), with access to personal protective equipment?				
	**Infection Prevention and Control**	YES	NO	I Don't Know	In Process
5.1.221	Are health care workers (HCWs), patients, and visitors are aware of respiratory and hand hygiene and the prevention of health care-associated infections?				
5.1.222	Is there a triage procedure in place in the emergency department for isolation of suspected and confirmed cases?				
5.1.223	Does the hospital Identify, sign and equip available areas for the medical care of suspected and confirmed cases in secure and isolated conditions?				
5.1.224	Are health workers trained in the use of personal protective equipment and consider additional precautions for specific transmission mechanisms (droplets, contact, aerosols, fomites)?				
5.1.225	Does the hospital ensure a 1-metre distance between beds regardless of whether patients are suspected of having COVID-19?				
5.1.226	Is there a limitation on visitors to those essential for patient support? Do visitors apply droplet and contact precautions?				
5.1.227	Is a record maintained of all people entering each patient’s room, including all staff and visitors?				
5.1.228	Does the hospital ensure strict supervision on the implementation of infection prevention and control measures?				
	**General**	YES	NO	I Don't Know	In Process
5.1.229	Is the CORE team of ER COVID is established?				
5.1.230	Did you receive training on PPE donning and doffing?				
5.1.231	Do you feel you have subsequent PPE available now?				

	Gloves				
	Masks				
	Surgical				
	N-95				
	Face shields/Goggles				
	Gowns				
